# Phlegmasia Alba Dolens—Death

**Published:** 1849-01

**Authors:** 


*Phlegmasia Alba Dolens—Death. Phlebitis with Obliteration of the Right Iliac Vein.*—Louise Prevot, a servant, aged twenty-two entered the hospital under the care of M. Trousseau. She had been confined a month ; her labour was very protracted, and it was necessary to apply the forceps in order to accomplish delivery. Five days afterwards she was seized with phlegmasia alba dolens, which was attended with fever, and the patient was obliged to keep her bed. At the time she entered the hospital there was œdematous tumefaction of the abdominal region on the right side. Pressure produced very severe pain at the posterior and superior part of the right leg. The abdomen was soft and inactive ; fever intense ; and depression considerable. Auscultation showed pneumonia of the right side through its whole extent, with pleuritic effusion at the inferior parts. Two bleedings were had recourse to ; the blood was very firm. Ipecacuanha was administered, and a pectoral tisane.

Although, in addition to these means, a large blister was applied to the back, the disease made rapid progress. The pneumonia increased, and, with it, the depression and fever. The pulse became small, compressible, and very frequent. The adynamic condition of the patient was most marked ; the skin became excoriated near the sacrum ; the respiration embarrassed more and more ; certain cerebral phenomena manifested themselves, and the patient died six days after her admission into the hospital.

The autopsy was made twenty hours after death. The right primitive iliac vein near its junction with the vena cava, the external iliac, the femoral, to within five or six centimètres of the crural arch, were completely obliterated. The femoral vein was pervious near the popliteal cavity. All the deep-seated veins of the right limb were obliterated. In the primitive iliac the obliteration was produced by a fibrinous mass, containing a notable quantity of a liquid, somewhat like pus, mixed with serum. The periphery of the stratum was composed of fibrinous matter to the extent of eight or ten centimètres, adherent to the neighbouring veins. At this point the veins were thickened and rigid, like that of a large artery, but without redness. Immediately beneath, a puriform liquid filled the vessel ; and still lower, the obliteration was caused by a large clot, composed partly of fibrine and partly of blood. The clots in the deep vein of the leg were formed in the same way.

In the thorax there was purulent effusion on the right side. Pneumonia on the same side, with small purulent spots scattered throughout. There was also peri-pneumonia of both lungs. The abdominal viscera were all healthy. No obliteration or any appreciable inflammation of the uterine cavity. No alteration of the internal surface of the uterus, or in the substance of the organ.—*Brit. Rec. of Obs. Med.*

